# Pharmacology of DB844, an Orally Active aza Analogue of Pafuramidine, in a Monkey Model of Second Stage Human African Trypanosomiasis

**DOI:** 10.1371/journal.pntd.0001734

**Published:** 2012-07-24

**Authors:** John K. Thuita, Michael Z. Wang, John M. Kagira, Cathrine L. Denton, Mary F. Paine, Raymond E. Mdachi, Grace A. Murilla, Shelley Ching, David W. Boykin, Richard R. Tidwell, James E. Hall, Reto Brun

**Affiliations:** 1 Trypanosomiasis Research Centre, Kenya Agricultural Research Institute (TRC-KARI), Kikuyu, Kenya; 2 Department of Pharmaceutical Chemistry, University of Kansas, Lawrence, Kansas, United States of America; 3 Eshelman School of Pharmacy, University of North Carolina at Chapel Hill, Chapel Hill, North Carolina, United States of America; 4 SVC Associates, Inc., Apex, North Carolina, United States of America; 5 Chemistry Department, Georgia State University, Atlanta, Georgia, United States of America; 6 Pathology Department, School of Medicine, University of North Carolina at Chapel Hill, North Carolina, United States of America; 7 Swiss Tropical and Public Health Institute and University of Basel, Basel, Switzerland; New York University School of Medicine, United States of America

## Abstract

Novel drugs to treat human African trypanosomiasis (HAT) are still urgently needed despite the recent addition of nifurtimox-eflornithine combination therapy (NECT) to WHO Model Lists of Essential Medicines against second stage HAT, where parasites have invaded the central nervous system (CNS). The pharmacology of a potential orally available lead compound, N-methoxy-6-{5-[4-(N-methoxyamidino) phenyl]-furan-2-yl}-nicotinamidine (DB844), was evaluated in a vervet monkey model of second stage HAT, following promising results in mice. DB844 was administered orally to vervet monkeys, beginning 28 days post infection (DPI) with *Trypanosoma brucei rhodesiense* KETRI 2537. DB844 was absorbed and converted to the active metabolite 6-[5-(4-phenylamidinophenyl)-furanyl-2-yl]-nicotinamide (DB820), exhibiting plasma C_max_ values of 430 and 190 nM for DB844 and DB820, respectively, after the 14th dose at 6 mg/kg qd. A 100-fold reduction in blood trypanosome counts was observed within 24 h of the third dose and, at the end of treatment evaluation performed four days post the last drug dose, trypanosomes were not detected in the blood or cerebrospinal fluid of any monkey. However, some animals relapsed during the 300 days of post treatment monitoring, resulting in a cure rate of 3/8 (37.5%) and 3/7 (42.9%) for the 5 mg/kg×10 days and the 6 mg/kg×14 days dose regimens respectively. These DB844 efficacy data were an improvement compared with pentamidine and pafuramidine both of which were previously shown to be non-curative in this model of CNS stage HAT. These data show that synthesis of novel diamidines with improved activity against CNS-stage HAT was possible.

## Author Summary

New drugs are needed to treat sleeping sickness, especially the second stage of the disease, which is characterised by the presence of parasites (trypanosomes) in the brain. The purpose of this work was to determine whether DB844, a new drug that is converted to the active form (DB820) after oral administration, has the potential to treat second stage sleeping sickness. Two dosing regimens of DB844 were evaluated in two groups of vervet monkeys that were infected experimentally with trypanosomes. Treatment was initiated four weeks after infection, when the monkeys were in second stage sleeping sickness, as confirmed by the presence of trypanosomes in brain fluid. Orally administered DB844 was well absorbed, tolerated and resulted in a decrease of trypanosomes in both the blood and brain fluid. However, some monkeys relapsed after treatment, with an overall cure rate of approximately 40% in both study groups. For at least two days after last dosing, the active drug, DB820, achieved blood concentrations known to be at least 19 times more than the minimum concentration that has been shown to be effective against a stringent human infective trypanosome isolate (STIB 900). These results represent an advance in efforts to develop new related compounds as oral treatments for sleeping sickness.

## Introduction

Human African trypanosomiasis (HAT, sleeping sickness) is a debilitating disease that is caused by the protozoan parasites, *Trypanosoma brucei gambiense* and *T. b. rhodesiense.* The disease is transmitted by tsetse flies (*Glossina spp*) and is therefore endemic only in geographical areas (foci) where both the parasite and vector are present; these foci are distributed in ∼20 sub-Saharan African countries and are home to at least 50 million people who are at risk of contracting HAT [1]. The spatial and temporal distribution of HAT is further determined by emergence of virulent parasite strains, breakdown in control and/or surveillance activities, changes in climate and vegetation and movements of carrier livestock species across borders [2]. As a result, the epidemiology of HAT is characterised by periodic epidemics interspersed with periods of near total eradication [3]. In 2009, the annual incidence dropped below 10,000 reported cases for the first time in 50 years, a success credited to the World Health Organization (WHO), national disease control programmes, bilateral co-operation and non-governmental organizations [4]. However, control activities must be maintained and new diagnostics and drugs developed to have a realistic chance of eventually eliminating HAT, a disease which has a history of reversing previous gains [2], [5]–[7].

Modern drug research and development activities for HAT have recently increased markedly, primarily through the efforts of public private partnerships (PPP's), which are funded by governmental and philanthropic organizations. A first success of these PPP's is the nifurtimox-eflornithine combination therapy (NECT), a product that has was recently added to the WHO essential medicines list for the management of *T. b. gambiense* CNS-stage infections [4], [8]. In addition, the dimethoxyamidine prodrug pafuramidine (DB289), a pentamidine-like compound developed by the Consortium for Parasitic Drug Development (CPDD), became the first oral drug to enter phase III clinical trials for 1^st^ stage HAT [9]. Clinical and preclinical investigations on pafuramidine (DB289) demonstrated that oral diamidine prodrugs could achieve efficacy equal to or better than pentamidine in the management of 1^st^ stage HAT [9]–[12]. However, the development program was terminated after some subjects (6%) in an extended phase I clinical trial (14 day dose regimen of 100 mg bid) developed delayed renal insufficiency [9]. Like pentamidine, pafuramidine did not achieve cure against 2^nd^ stage HAT in animal models [11], [12].

In an effort to develop a compound that was well tolerated and with efficacy against 2^nd^ stage HAT, a next-in-class dimethoxyamidine prodrug, N-methoxy-6-{5-[4-(N-methoxyamidino) phenyl]-furan-2-yl}-nicotinamidine (DB844), was evaluated. Similar to the biotransformation of DB289, DB844 was shown to be sequentially *O*-demethylated and *N*-dehydroxylated in human liver microsomes to form its active metabolite, 6-[5-(4-phenylamidinophenyl)-furanyl-2-yl]-nicotinamide (DB820) [13]. In mice, oral DB844 appeared to be well absorbed and converted to DB820, and cured all animals (5/5) in the GVR35 CNS model of HAT [11]. In vitro, DB820 was a potent trypanocide with an IC_50_ value of 2.4 ng/ml (5.2 nM) against *T. b. rhodesiense* STIB900 [11]. DB820 also accumulated in the DNA containing organelles and bound to DNA molecules preferentially at AT rich sites, thus likely sharing the same mechanism of action with pentamidine [14]–[16]. The purpose of this study was to further evaluate the potential of DB844 as a novel oral treatment against 2^nd^ stage HAT by characterizing its pharmacology in vervet monkeys, a species commonly used as a preclinical model for HAT [12]. The specific study objectives were to (a) characterize the metabolic profile of the prodrug in vervet monkey liver microsomes; (b) evaluate the toxicity of orally administered DB844 in un-infected monkeys to understand tolerability and to define an appropriate dose range for efficacy studies in the monkey HAT model; and (c) evaluate the pharmacokinetics, efficacy and safety of DB844 in the infected vervet monkey model, which closely mimics human sleeping sickness.

## Materials and Methods

### Ethics Statement

Studies were undertaken in adherence to experimental guidelines and procedures approved by the Institutional Animal Care and Use Committee (IACUC), the ethical review committee for the use of laboratory animals at the Trypanosomiasis Research Centre of the Kenya Agricultural Research Institute (TRC-KARI). The experimental guidelines also complied with National guidelines of the Kenya Veterinary Association.

### Trypanocidal Test Compound

**Figure 1:**
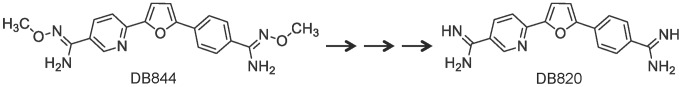
Chemical structure of DB844 and DB820.

The test compound N-methoxy-6-{5-[4-(N-methoxyamidino) phenyl]-furan-2-yl}-nicotinamidine (DB844) (Figure 1) was synthesized in the laboratory of Dr. David Boykin (Georgia State University, Atlanta, GA, USA) as previously reported [17]. The current study used DB844 (lot D, C_19_H_19_N_5_O_3_·3HCl·H_2_O) with a purity of >95% as determined by both NMR [17] and high performance liquid chromatography (HPLC)/UV (described below). DB844 was supplied to KARI-TRC through CPDD in the form of yellow powder in opaque and water tight bottles. Once received, the drug containing bottle was wrapped in aluminium foil and refrigerated at 4–8°C. Dosing formulations were prepared daily by dissolving the drug in de-ionised distilled water to render concentrations of 5 and 6 mg/ml. Reconstituted drug was protected from light by wrapping drug-containing vials with aluminium foil.

### Trypanosome Isolate

A pleomorphic isolate, *T.b rhodesiense* KETRI 2537, a derivative of EATRO 1989 that was isolated from a patient in Uganda by direct inoculation of blood and lymph node aspirate into a monkey and later cryopreserved at KARI- TRC [18], was used. This isolate is the basis of the KETRI vervet monkey and mouse models and has been widely used for drug efficacy trials [19], [20].

### Experimental Animals

Adult vervet monkeys [*Chlorocebus (Cercopithecus) aethiops* syn. African green monkeys] (n = 22) weighing between 2.5 and 5.5 kg were acquired from the Institute of Primate Research (IPR) in Kenya. Monkeys were quarantined, screened, acclimated for the study for a minimum of 90 days while being screened for evidence of disease as previously described [12], [20], [21]. They were also de-wormed, treated for ectoparasites, and acclimated to staying in individual squeeze-back stainless steel cages and human handling. The monkeys were fed a diet of fresh vegetables and commercial monkey cubes (Unga feeds, Nakuru Kenya) twice daily and provided water *ad libitum.*

### Metabolism of DB844 in Vervet Monkey Liver Microsomes

The metabolism of DB844 was investigated using liver microsomes, prepared from a male vervet monkey by XenoTech, LLC (Lenexa, KS), in the presence of NADPH as described previously [22] with modifications. Briefly, incubation mixtures (1 ml at pH7.4, in triplicate) contained 10 µM DB844 and 0.2 mg/ml monkey liver microsomes. After a 5-min equilibration period at 37°C, reactions were initiated with the addition of NADPH. Aliquots (50 µl) of the reaction mixtures were removed at 0, 5, 10, 15, 30, 60, 90, and 120 min and mixed with 25 µl of ice-cold acetonitrile. The mixtures were centrifuged (10,000×g for 5 min at 4°C) and the supernatants were analyzed by HPLC/UV using the same method as previously described for DB289 [22]. Metabolite identification was performed by comparing retention times to those of synthetic standards, which include M1A (DB1284), M1B (DB1058), M2A (DB1285), M2B (DB1212), M3 (DB821), and DB820 [13]. DB844 and metabolites were quantified using a single-concentration calibration curve generated using synthetic standards.

### Toxicity Study in Uninfected Monkeys

The toxicity of DB844 was evaluated in uninfected vervet monkeys with the aim of defining the appropriate dose-range for the compound, identifying target organs of toxicity and characterizing the nature of drug-induced toxicity in this species. Six monkeys were used to evaluate the tolerability of 10-day oral dose regimens. Baseline clinical and haematology data were collected during a 14-day period, after which two monkeys (one male and one female) per dose group were administered with DB844 at 5, 10 or 20 mg/kg/day for 10 days via oral gavage. A dose volume of 1 ml/kg was administered. Daily ward rounds were conducted to assess feed intake (appetite), demeanour, posture and stool composition and consistency. Feed intake was assessed by scoring the proportion of the daily ration consumed by each monkey based on a scale of 1 (full ration eaten), 3/4, 1/2, 1/4 and 0 (no feed eaten) as previously described [21].

Monkeys were monitored for 28 days post dosing. They were anaesthetized through intramuscular (IM) injection with ketamine HCl (10–15 mg/kg) and Valium® (0.5 mg/kg) to facilitate physical examination, body weight measurements and sample collection. Blood was collected from the femoral vein *via* inguinal venipuncture as described previously [20], [23] and divided into aliquots: 1 ml in EDTA for full haemogram determination and 2 ml in EDTA for plasma separation. Plasma was separated using a cool spin centrifuge (4°C, 1500 revolutions/minute), separated into aliquots and stored at −20°C pending analysis for DB844/DB820 concentrations.

When overt drug related toxicity was detected, drug administration was withdrawn from the affected monkey(s) to allow the affected individuals to recover. Monkeys that failed to recover were humanely euthanized using 20% pentobarbitone sodium (Euthatal®, Rhone Merieux) for gross and histopathology examination. Euthanasia was carried out when monkeys were judged to have deteriorated to the *in extremis* condition, characterised by inability or reluctance to perch and very low feed intake (less than 1/4 of daily ration) for 2–3 consecutive days [21], [24]. Organ specimens from these monkeys were preserved in 10% formalin and later sectioned for histopathogy. The processed slides were stained with haematoxylin and eosin.

### Monkey Infections

Sixteen vervet monkeys in two groups of eight monkeys each (four males and four females) were used. After a 14-day baseline weight, clinical and haematology data collection period, the sixteen monkeys were infected by intravenous injection of approximately 10^4^ trypanosomes diluted from infected blood of immuno-suppressed donor Swiss white mice [12]. Parasitaemia post infection was determined by examination of wet film of ear prick blood and/or examination of buffy coat after centrifugation of blood collected in a heparinised capillary tube as described previously [25]. Parasitaemia in wet film was estimated using the rapid matching method of Herbert and Lumsden [26]. In addition, monkeys were confirmed to be in second stage disease by detection of trypanosomes in the CSF with or without elevated white cell counts above 5 cells/µl [4], [20], [21], [23]. At 28 days post infection (DPI), the animals were treated with DB844 via oral gavage at 5 mg/kg qd×10 days (group I, n = 8) or 6 mg/kg qd×14 days (group II, n = 8), utilising a dose volume of 1 ml/kg. The monkeys were examined for parasitaemia every day during drug treatment and then twice weekly until ≥300 days post dosing, at which point monkeys were considered cured if they remained clinically normal and parasite-free as determined below.

### Pre and Post Treatment Monitoring

Daily ward rounds were conducted throughout the study to assess feed intake, demeanour, posture, and stool composition and consistency. Feed intake was assessed by scoring the proportion of the daily ration consumed by each monkey on a scale of 1 (full ration eaten), 3/4, 1/2, ¼, and 0 (no feed eaten) as previously described [21]. Monkeys were anaesthetised weekly by intramuscular injection of ketamine HCl (10–15 mg/kg) and Valium® (0.5 mg/kg) for physical examination, body weight measurements and collection of whole blood in EDTA and cerebrospinal fluid (CSF) samples. Blood samples (1 ml) were thereafter collected for preparation of plasma for pharmacokinetic studies at 1, 2, 4, 8, 24, 48, 96 and 168 h and then weekly until 28 days, while CSF samples (0.7–1.5 ml) were collected at 1, 24, 96, 168 h and then weekly until 28 days post last dosing. Samples were collected by inguinal venipuncture (blood) or lumbar puncture (CSF) of anaesthetised monkeys as previously described [12]. Plasma was separated using a cool spin centrifuge (4°C, 1500 RPM). After 28 days post last dosing, blood and CSF samples were collected once every two weeks up to 100 days, then once per month until 300 days post dosing for haematology and parasite detection only. During sampling, some of the free-flowing CSF was collected into a capillary tube and immediately transferred onto a haemocytometer (Neubaeur) chamber for counting of trypanosomes and/or white blood cells. Samples that were negative for CSF trypanosomes by direct microscopy were concentrated and examined according to the modified single centrifugation technique [21], [27]. All the CSF samples that remained negative for trypanosomes after the concentration step were then sub-inoculated into Swiss white mice (2 mice per sample) to further aid in diagnosis of infected fluids. Similarly, blood samples that were negative for trypanosomes after concentration [26] were inoculated into Swiss white mice. When trypanosomes were detected in blood and/or CSF or when monkeys were diagnosed to have attained *in extremis* condition as previously described [21], [24], they were humanely euthanized using 20% pentobarbitone sodium (Euthatal®, Rhone Merieux) for post mortem examination.

Haematology samples (1 ml) were analysed using an AC^3diff^T Coulter Counter (Miami, Florida, USA). Clinical chemistries were determined using a Humalyzer analyser system. Finally, plasma and CSF were analyzed for drug and metabolite concentrations using an HPLC-tandem Mass Spectrometry (HPLC-MS/MS) procedure as described below.

### Sample Preparation and HPLC-MS/MS Quantification

Monkey plasma and CSF samples were prepared and quantified for DB844 and DB820 using previously described methods [22], [28] with modifications. Briefly, plasma or CSF samples (25 µl) were extracted with 200 µl of 7∶1 (v/v) methanol∶water containing 0.1% (v/v) trifluoroacetic acid and deuterated internal standards (30 nM each for DB844-d4 and DB820-d4), followed by centrifugation, evaporation, and reconstitution before HPLC-MS/MS analysis [28]. HPLC-MS/MS quantification of DB844 and DB820 was performed on an Applied Biosystems (Foster City, CA) API 4000 triple quadruple mass spectrometer equipped with a Turbo IonSpray interface in positive ion mode (MDS Sciex, San Francisco, CA). Reconstituted samples (4–5 µl) were separated on an Aquasil C18 analytical column 2.1×50 mm, 5 µm (Thermo Electron, Waltham, MA) with mobile phases consisting of HPLC-grade water containing 0.1% formic acid (A) and methanol containing 0.1% formic acid (B). After a 0.4-min initial hold at 15% B, mobile phase composition began with 15% B and was increased to 80% B over 1.6 min, followed by a 1.0-min hold, at a flow rate of 0.5 ml/min. The column was then washed with 95% B for 1.3 min at a flow rate of 0.5 ml/min and was re-equilibrated with 15% B at a flow rate of 0.5 ml/min for 0.5 min before injection of the next sample. The characteristic SRM transitions for DB844 and DB820 were m/z 366.2→319.2 and 306.2→289.2, respectively. The calibration curves for DB844 ranged from 2.5–2500 nM and 1–1000 nM in plasma and CSF, respectively, using a quadratic equation with 1/x weighting. The calibration curves for DB820 ranged from 10–2500 nM and 1–1000 nM in plasma and CSF, respectively, using a quadratic equation with 1/x weighting.

### Data Analysis

Data were analysed statistically using Statview for Windows Version 5.0.1 (SAS Institute Inc, Cary, NC). Repeated measures ANOVA, with Fishers PLSD post hoc test, was used to test the effects of trypanosomal infection, as well as DB844, on haematology and clinical chemistry parameters in comparison with respective baseline values (α = 0.05). Confidence intervals [95%] were derived to further test the significance of observed findings. Pharmacokinetic outcomes were determined using standard non-compartmental methods performed using Phoenix WinNonlin (version 6.2, Pharsight, Mountain View, CA).

## Results

### Metabolism Profiles in Monkey Liver Microsomes

**Figure 2:**
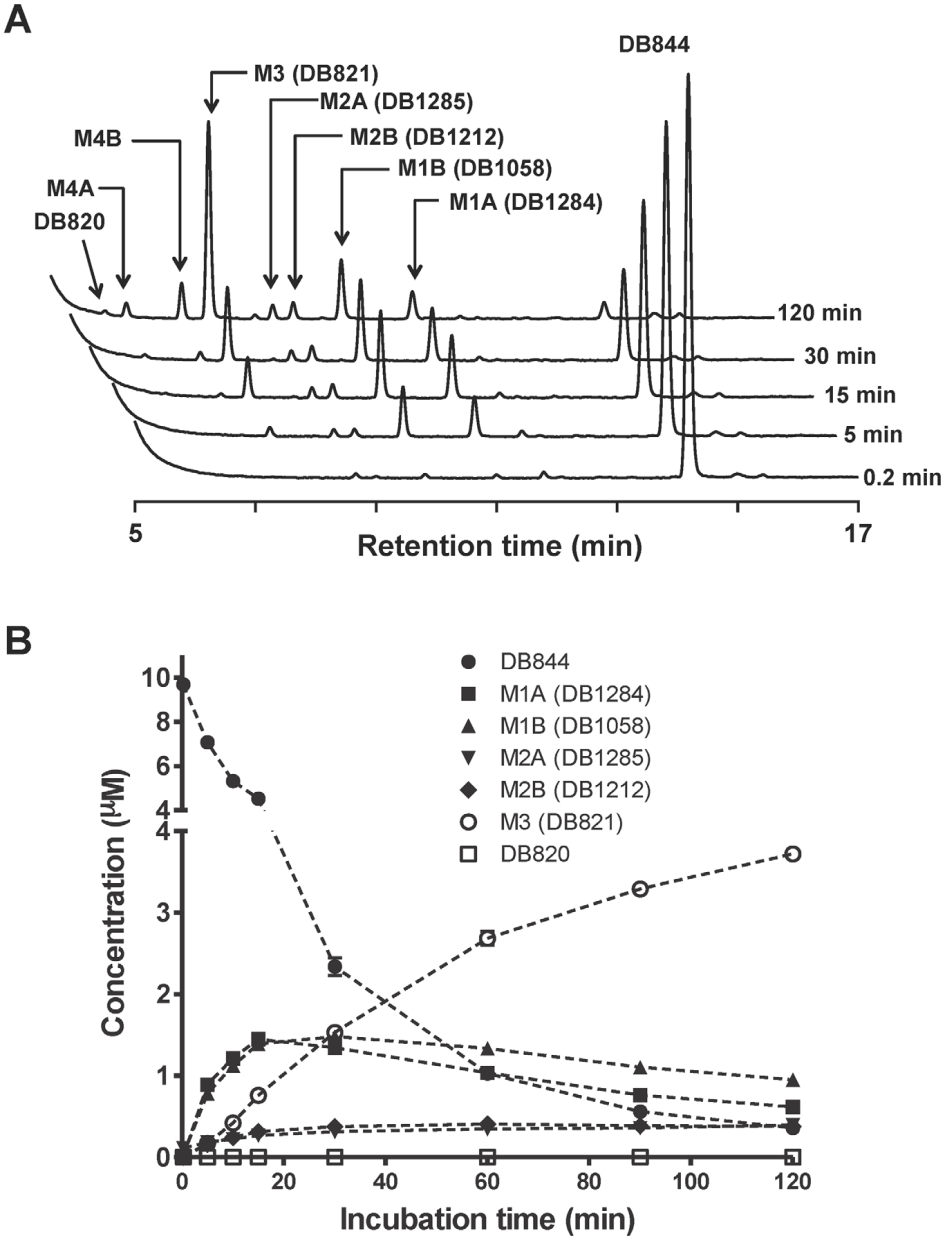
HPLC/UV chromatograms and concentration-time profiles of DB844/metabolites following incubation of DB844 with male vervet monkey liver microsomes. A: HPLC/UV chromatograms; B: Concentration-time profiles of DB844 and metabolites. Incubation mixtures (1 ml at pH7.4, in triplicate) contained 10 µM DB844 and 0.2 mg/ml monkey liver microsomes. Aliquots were taken at 0.2, 5, 15, 30, and 120 min and evaluated for concentrations of DB844 and six metabolites (M1A, M1B, M2A, M2B, M3, and DB820). Metabolites M4A and M4B were not quantified due to lack of synthetic standards.

DB844 was rapidly metabolized in vervet monkey liver microsomes (MLM) with a microsomal half-life of approximately 14 min to form at least seven metabolites over a 120 min incubation period (Figure 2). The first two metabolites to be detected, M1A and M1B, were likely formed through the oxidative *O*-demethylation of either the pyridyl or phenyl side of DB844 [13]. M1A and M1B gave rise to M2A and M2B respectively, through reductive *N*-dehydroxylation, or further O-demethylation to form the bis-amidoxime metabolite, M3. The *O*-demethylation of M2A and M2B resulted in M4A and M4B, respectively, which could also be generated by *N*-dehydroxylation of M3. At last, the *N*-dehydroxylation of M4A and M4B gave rise to the active metabolite DB820 (Figure 2). Metabolites M1A and M1B attained the highest concentrations during the initial 20 minutes of incubation after which M3 became the metabolite with the highest concentration in the drug/liver microsome mixture (Figure 2).

### Toxicity

Uninfected monkeys were orally dosed with DB844 at 5, 10 or 20 mg/kg/day for 10 days. The lowest, 5 mg/kg, did not elicit adverse clinical signs of toxicity in 2/2 monkeys (Table 1). Overt toxicity was however observed in 1/2 and 2/2 monkeys to which DB844 was administered at 10 and 20 mg/kg, respectively (Table 1). In the high dose group, overt toxicity was confirmed, at the earliest, after eight daily doses (cumulative dose [CD] = 160 mg/kg). Drug administration to these monkeys was immediately withdrawn to allow clinical recovery which, however, did not occur. As a result, the monkeys were humanely euthanized 1–2 days later (9–10 days post first dosing) (Table 1). Both monkeys from the middle (10 mg/kg) dose group completed the 10-day dose regimen successfully (CD = 100 mg/kg) after which 1/2 developed signs of overt toxicity and was eventually euthanized 16 days post first dosing (Table 1). The adverse clinical events included anorexia, gastrointestinal disturbances (vomiting or changes in stool consistency), jaundice and weight loss of up to 10.5% (Table 1). Haematology revealed nothing significant except for vervet 578 (10 mg/kg); in this monkey, the red cell distribution width (RDW) rose from 15 to 18.4 (23%) while mean corpuscular volume (MCV) rose from 76.6 to 86.9 fl (13%). At histopathology examination, lesions observed included inflammation and erosions of the gastrointestinal tract (GIT), fatty change (steatosis) in the liver, hydropic degeneration of renal tubular cells and haemorrhage and haemosiderosis in multiple organs (Table 1). Toxicokinetic analysis revealed that in the two monkeys that were dosed at 5 mg/kg, DB844 and DB820 achieved average concentrations of 215 nM and 41.6 nM respectively at 1 h post last dosing. However, other toxicokinetic measurements could not be determined due to the limited sampling.

**Table 1:** Safety of oral DB844 dose regimens in un-infected vervet monkeys.

		I: DB 844 at 5 mg/kg×10 days orally		II: DB844 at 10 mg/kg×10 days orally		III: DB844 at 20 mg/kg×10 days orally	
Parameters evaluated		572F	582M	541F	578M	543F	606M
Adverse clinical events	Reduced feed intake	None	None	Yes (11)	Yes (14)	Yes (8)	Yes (9)
	GIT changes	None	None	None	Yes (16)	Yes (8)	Yes (8)
	Jaundice	None	None	None	yes	yes	yes
	% weight loss	1.8	4.9	4.6	10.5	9.1	6.5
	Daily doses completed	10/10	10/10	10/10	10/10	8/10	8/10
	Euthanised due to toxicity	No	No	No	Yes (16)	Yes (9)	Yes (10)
Liver histology	Fatty change (Steatosis)	NA	NA	NA	+++	+++	+++
	Inflammation	NA	NA	NA	++	++	+++
	Focal necrosis	NA	NA	NA	+++	+++	+++
	Haemosiderosis	NA	NA	NA	++	+	+++
GIT histology	Ulcers/erosions	NA	NA	NA	+++	+++	+++
	Inflammation	NA	NA	NA	+++	+++	+++
	Haemosiderosis	NA	NA	NA	++	+	+++
Spleen histology	Expanded red pulp	NA	NA	NA	+++	++	++
	Haemosiderosis	NA	NA	NA	+++	+++	+++
Kidney histology	Hydropic degeneration/interstitial oedema	NA	NA	NA	+++	+	+

Key: GIT = gastrointestinal system; numbers in parenthesis = time in days post first drug dose when an adverse clinical event was observed; NA = not assessed since the monkeys were not euthanized; F = female; M = male.

### Progression of the *T.b. rhodesiense* Infection

**Figure 3:**
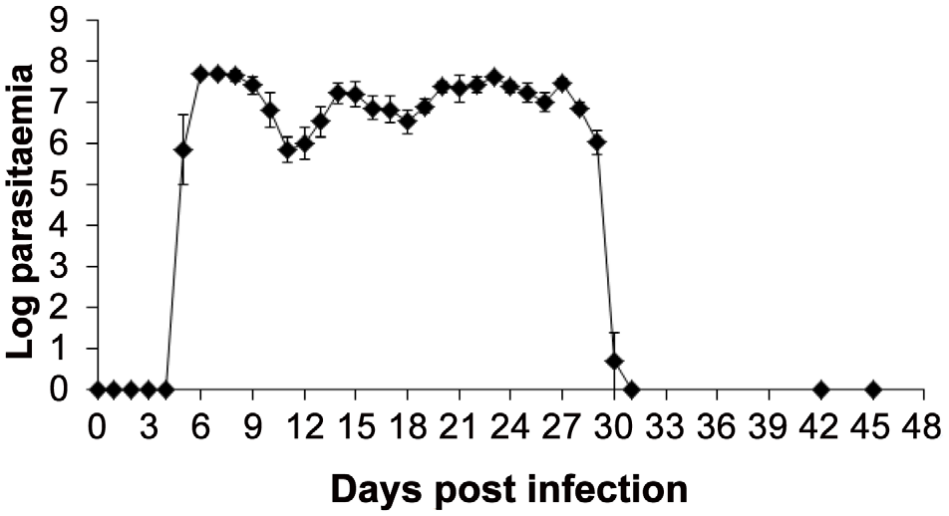
Parasitaemia pattern in monkeys infected with *T.b. rhodesiense* KETRI 2537 and subsequently treated with DB844. Symbols and error bars represent means and SEs, respectively, of 7 animals; monkeys were treated with DB844 at 6 mg/kg×14 days, from 28–41 days post infection; Log parasitaemia values were determined by microscopic examination of wet smears of blood using the matching method of Herbert and Lumsden, 1976 [23].

The pre-patent period of the experimental *T. b. rhodesiense* infection in both groups of monkeys was approximately 5–6 days (Table 2). Parasitaemia rose to a peak of 5.0×10^7^ trypanosomes (antilog 7.7) within 2–3 days (7–8 DPI) but subsequently fluctuated to give characteristic waves of parasitaemia (Figure 3). Monkeys developed a classical *T. b. rhodesiense* clinical disease characterised by reduction in feed intake, raised hair coats, reduced activity, dullness and/or excitability when the clinical signs were first observed at 4–7 DPI. Anorexia and inactivity were transient, lasting a maximum of 3 days before normal appetite and activity were regained. Enlargement of peripheral lymph nodes (especially axillar and inguinal lymph nodes) and splenomegaly (up to 3 times compared to pre-infection) were also observed 7–14 DPI while facial, scrotal or eyelid oedema were observed in 8/16 (50%) of the infected monkeys from 20 DPI. Average (± SE) weight before infection was 3.6±0.4 (range = 2.6–5.5) and 3.1±0.3 (range = 2.3–4.2) kg for group I and II, respectively. Four weeks after infection, weight decreased significantly (p = 0.0003) by 6.4% and 5.6%, respectively. Time to parasitization of the cerebrospinal fluid (CSF) was a median 21 days (range = 7–27) for both groups of monkeys (Table 2). At 27 DPI, one day before initiation of treatment with DB844, trypanosome numbers ranged from 1–8/µl of CSF; median cell numbers were 5.0 (range = 0–45) and 6.0 (range = 1–20) cells/µl of CSF in groups I and II respectively. During previous weekly samplings, white cell counts in some monkeys increased to 152/µl of CSF (Table 2).

**Table 2:** Treatment outcome in monkeys treated with DB844 while in second stage *T. b. rhodesiense* infection.

Group	Monkey ID	PP (DPI)	Time to CSF parasitization (DPI)	Tryps/µl CSF at 27 DPI	WC/µl of CSF at 27 DPI	EoT test at 1 day post last dose	EoT test at 4 days post last dose	TOC test at 300 days post last dose
I	566	5	7	1	4 [17]	Neg	Neg	Cured
	568	5	27	1	45 [45]	Neg	Neg	Cured
	576	5	21	1	2 [12]	Neg	Neg	Relapsed
	599	6	14	8	39 [152]	Pos	Neg	Relapsed
	601	6	21	1	0 [6]	Neg	Neg	Relapsed
	603	6	21	1	6 [44]	Neg	Neg	Relapsed
	607	5	14	2	1 [28]	Neg	Neg	Relapsed
	609	6	21	1	8 [8]	Neg	neg	Cured
Median (range)		5.5 (5–6)	21 (7–27)	1(1–8)	5 (0–45)	Cure rate = 7/8 (88%)	Cure rate = 8/8 (100%)	3/8 (37.5%)
II	571	6	21	1	4 [11]	Neg	Neg	Cured
	596	5	7	1	8 [19]	Neg	Neg	Cured
	600	5	14	1	6 [6]	Neg	Neg	Cured
	624	5	7	1	4 [4]	Pos	Neg	Relapsed
	625	5	21	1	1 [20]	WD		
	630	5	21	4	20 [20]	Pos	Neg	Relapsed
	652	5	21	2	10 [11]	Neg	Neg	Relapsed
	653	5	28	2	6 [141]	Neg	Neg	Relapsed
Median (range)		5.0 (5–6)	21 (7–27)	1 (1–4)	6 (1–20)	Cure rate 5/7 (71%)	7/7 (100%)	3/7 (42.5%)

Key: ID = identity in the laboratory; PP = pre-patent period; DPI = days post infection; CSF = cerebrospinal fluid; Tryps = trypanosomes; WC = white cells; EoT = end of treatment; ToC = test of cure; Neg = Negative; Pos = positive; WD = withdrawn from the experiment after 10^th^ drug dose due to toxicity; Numbers in square brackets = maximum number of white cell counts observed during any of the four weekly samplings between 0–27 DPI; I: DB844 5 mg/kg×10 days per os; 28–37 DPI; II: DB844 6 mg/kg×14 days per os; 28–41 DPI.

### Efficacy

At 28 DPI when monkeys had shown characteristic features of 2^nd^ stage infection (i.e., presence of trypanosomes and elevated white blood cell counts above 5/µl of CSF), they were treated with DB844 at 5 mg/kg×10 days (group I) or 6 mg/kg×14 days (group II). At 24 h post third drug dose (i.e., 4^th^ day of treatment), trypanosomes were not detected in wet smears of peripheral blood (Figure 3), showing that at least 100-fold reduction in parasitaemia had been achieved, from 10^7^ to 10^5^ trypanosomes/ml of blood which is the detection limit of the matching method of Herbert and Lumsden [23]. End of treatment (EoT) evaluation was conducted at 1 and 4 days post last dosing time points. At the one day post dosing time point, trypanosomes were detected in some monkeys using sensitive trypanosome concentration techniques for both blood and CSF [25], [27] as evidenced by provisional cure rates of 7/8 (group I) and 5/7 (group II) (Table 2). The three monkeys with persisting low numbers of trypanosomes in the blood and/or CSF eventually tested negative at the 4 days post dosing time point (Table 2), demonstrating an EoT provisional cure rate of 100% for both groups.

Post treatment follow-up was carried out for at least 300 days post dosing. During this follow-up period, nine monkeys relapsed demonstrating an overall test of cure rate of 3/8 (37.5%) and 3/7 (42.5%) for the 5 mg/kg and 6 mg/kg dose groups respectively (Table 2). Trypanosomes were observed exclusively in the CSF in five of the relapsed monkeys. In three of the four remaining relapse cases, trypanosomes were detected in the CSF earlier than in the blood. Overall, the median (range) time to trypanosome recrudescence was 133 (35–322, n = 9) days for CSF and 261 (239–322, n = 3) days for blood trypanosomes. Despite trypanosomes becoming cleared from the peripheral blood by eighth day of dosing, monkey 625 (Table 2) developed toxicity and was humanely euthanized 2 days after administration of 10^th^ dose of DB844 at 6 mg/kg (CD = 60 mg/kg). At post mortem examination, liver and gastrointestinal toxicity were observed, comparable to findings in 1/2 (10 mg/kg) and 2/2 (20 mg/kg) un-infected monkeys euthanized due to DB844 toxicity.

### Haematology Changes in the Infected Monkey Model

**Figure 4:**
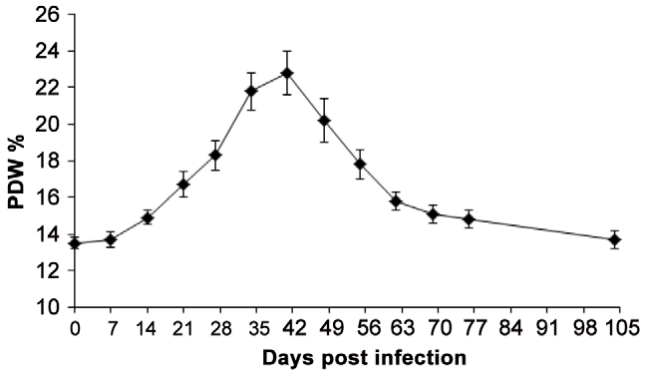
Changes in red cell distribution width in monkeys following infection and subsequent treatment with DB844. Symbols and error bars represent means and SEs, respectively, of seven monkeys that were treated with DB844 at 6 mg/kg×14 days, from 28–41 days post infection.

The trypanosome infection provoked a reduction in erythrocytes (red blood cells, RBC) and associated parameters. Average haemoglobin concentration declined by 32.1% in group II monkeys, from 13.4±0.6 [95% CI = 12.4–15.1] g/dl at baseline (day 0) to 9.1±0.6 [95% CI = 7.9–10.3] g/dl (p<0.0001] at 27 DPI (Table 3). Erythrocyte counts and haematocrit concentration, declined significantly (p<0.0001, Table 3) by 28.7% and 32.1%, respectfully. Mean corpuscular volume (MCV) and mean corpuscular haemoglobin (MCH) decreased significantly (p<0.0001) (Table 3). Erythrocyte associated parameters of group I monkeys exhibited similar trends (data not shown), indicating that the infection caused a microcytic hypochromic type of anaemia. Monkeys also experienced significant thrombocytopenia and leucopaenia (Table 3) related to the experimental *T. b. rhodesiense* infection. Upon treatment with oral DB844, no drug related haematology changes were observed. Trypanosome induced anaemia, thrombocytopenia and leucopaenia resolved rapidly. Baseline white cells numbers were re-established by end of treatment while platelet and RBC associated parameters were re-established within seven and 28–63 days respectively (Table 3; Figure 4).

**Table 3:** Haematologic effects of *T. b. rhodesiense* KETRI2537 infection and treatment with DB844 in vervet monkeys.

Parameters	Days post infection (days post last drug dose)					
	0	27	41 (0)	48 (7)	69 (28)	104 (63)
RBC (×10^6^/µl)	5.8±0.2	4.2±0.2, p<0.0001	4.8±0.3, p<0.0001	4.9±0.2, p<0.004	5.5±0.2, p = 0.55	6.1±0.3 p = 0.02
Hemoglobin (g/dl)	13.4±0.6	9.1±0.6, p<0.0001	11.2±0.7, p<0.0001	11.5±0.6, p<0.0001	12.8±0.5, p = 0.15	14.1±0.8, p = 0.11
Haematocrit %	43.9±2.0	29.8±1.7, p<0.0001	31.6±1.6, p<0.0001	31.8±1.6, p<0.0001	43.8±1.6, p = 0.91	46.7±2.7, p = 0.91
Mean corpuscular volume (fl)	78.2±1.5	70.8±0.8, p<0.0001	65.3±0.9, p = 0.0001	64.8±0.9, p = 0.0001	79.8±1.4, p = 0.11	77.0±1.5, p = 0.21
Mean corpuscular haemoglobin (g/dl)	23.9±0.4	21.6±0.3, p<0.0001	23.0±0.3 p<0.008	23.3±0.4 p = 0.07	23.4±0.6, p = 0.13	23.3±0.5 p = 0.21
Platelet counts (×10^3^/µl)	346.5±13.1	183.5±24.6 p = 0.002	406.5±22.5 p = 0.05	390.7±32.4, p = 0.11	332.2±11.9, p = 0.78	294.3±17.4 p = 0.55
WBC counts (×10^3^/µl)	5.8±0.5	3.0±0.4, p = 0.003	7.1±1.0, p = 0.12	5.6±1.2, p = 0.9	5.9±0.8, p = 0.8	5.1±0.1, p = 0.4

Key: RBC = red blood cells; WBC = White blood cells; g/dl = grams/decilitre; fl = femtolitres; p-values<0.05 indicate values that were significantly different from pre-infection baseline (day 0) values (Repeated measures Anova with Fishers PLSD post hoc test); Monkey were treated with DB844 at 6 mg/kg×14 days, from 28–41 days post infection.

### Clinical Chemistries

**Figure 5:**
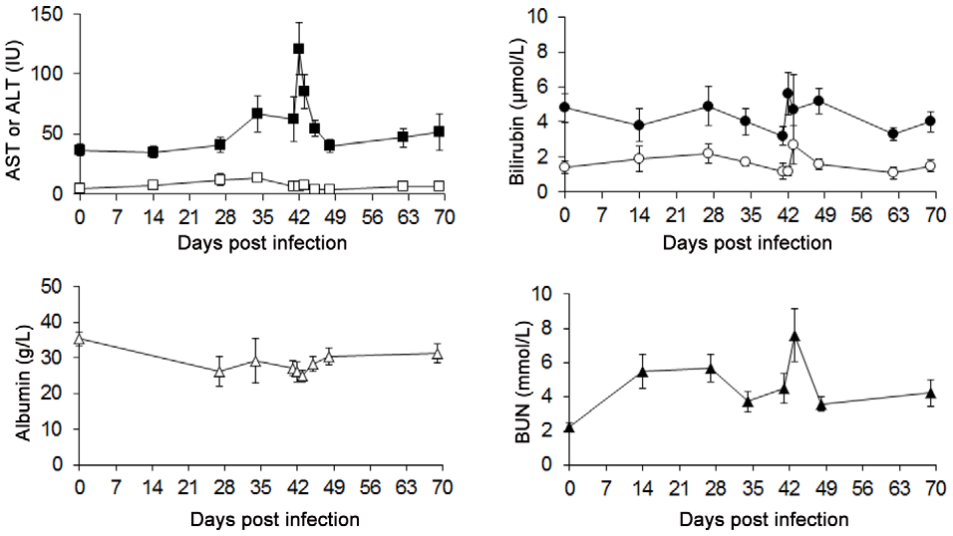
Transient infection and DB844 induced changes in clinical chemistry indicators of liver and kidney function. Symbols represent mean ± SE (n = 7) of aspartate aminotransferase (AST, ▪), alanine aminotransferase (ALT, □), total bilirubin (•), direct bilirubin (0), blood urea nitrogen (BUN, ▴) and albumin (◊); monkeys were treated with DB844 at 6 mg/kg×14 days, from 28–41 days post infection.

Plasma from infected monkeys treated with DB844 at 6 mg/kg (group II) was analyzed for several biomarkers of liver and kidney function. Plasma aspartate aminotransferase (AST) did not change significantly following infection but peaked transiently during drug administration (Figure 5, Figure S1). At 24 hours post dosing, mean (± SE) plasma AST increased to 3.3 times above baseline, from 37.0±4.8 IU [95% CI = 26.7–46.8] (day 0) to 121±21.6 IU [95% CI = 65.5–176.5, p = <0.0001]. Mean plasma alanine aminotransferase (ALT) exhibited an increasing trend immediately after infection (day 0) and peaked after 7 daily drug doses (34 DPI) (Figure 5). At its peak, mean plasma ALT increased by 2.7 times above baseline, from 4.5±0.9 IU [CI = 2.1–6.9] (day 0) to 13.6±3.8 IU [CI = 3.8–23.4; p = 0.008]. Aberrations in ALT resolved rapidly after treatment. Monkeys further demonstrated a significant infection-related 25.8% decrease in mean plasma albumin concentration, from 35.3±1.9 [CI = 30.4–40.3] g/l at baseline to 26.2±4.2 [95% CI = 15.4–37.0, p = 0.02] g/l at 27 DPI. Plasma albumin concentration stabilized during the treatment period, then decreased transiently to a nadir at 48 h post dosing (43 DPI, Figure 5). Total bilirubin and direct bilirubin concentrations fluctuated in a pattern comparable to the transaminases (Figure 5), but none of the changes were statistically significant. Mean alkaline phosphatase was 6.3±1.9 at baseline and 4.1±0.8 at 27 DPI (p = 0.25) and showed no significant changes both during and after drug administration. Two indicators of renal function, blood urea nitrogen (BUN) and creatinine, were evaluated. BUN indicated a mild and reversible decrease in kidney function from 48 h post dosing (43 DPI) (Figure 5). Mean plasma concentration of creatinine fluctuated in a similar pattern to BUN, but the change from baseline was not significant (data not shown).

### Pharmacokinetics

**Figure 6:**
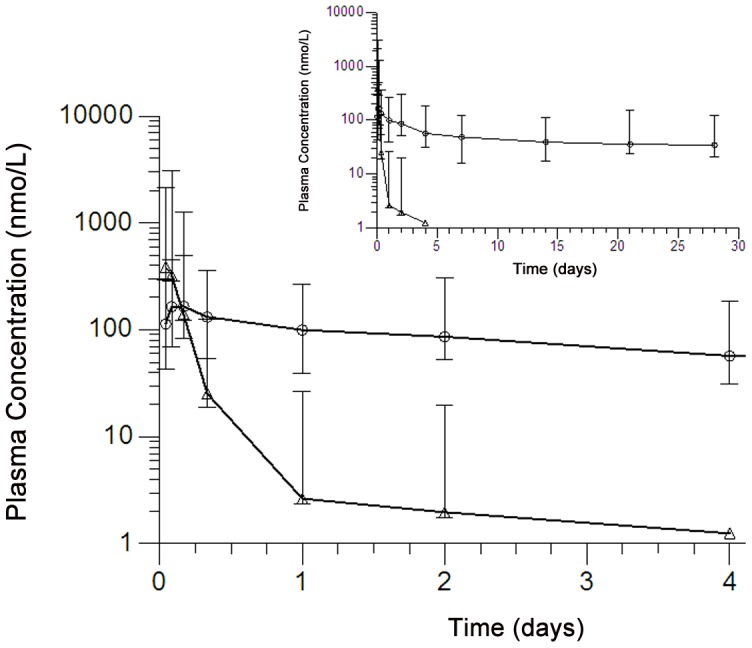
Plasma concentration-time profiles following oral administration of the last (14^th^) daily dose of DB844. Symbols and error bars represent geometric means and SEs, respectively, for DB844 (△) and DB820 (○). The monkeys (n = 7) were treated with DB844 at 6 mg/kg×14 days, from 28–41 days post infection. The insert graph shows the extended profile up to 28 days post the last daily dose of DB844.

Plasma and CSF collected from group II monkeys (6 mg/kg) were analyzed for various pharmacokinetic outcomes using traditional non-compartmental methods. After the last (i.e. 14^th^) dose of DB844, geometric mean (90% CI) concentrations of DB844 peaked at 1 h in plasma with a C_max_ of 430 (100–1800) nM (Table 4, Figure 6) with modest individual animal variations in PK profiles (Figure S2). The active metabolite, DB820, peaked at 4 h in plasma with a C_max_ of 190 (110–320) nM. Exposure to DB820 was three-fold higher than that of DB844, as assessed by the metabolite∶parent AUC ratio (Table 4). DB844 concentrations decreased at a faster rate than DB820 concentrations, with a geometric mean (90% CI) apparent terminal elimination half-life of 5.8 (3.4–9.6) h. DB820 was detected in plasma up to 28 days after the last dose of DB844 with a geometric mean (90% CI) concentration of 35 (14–86) nM. Given the protracted decline in DB820 plasma concentrations, the duration of plasma collection was insufficient for accurate estimation of the terminal half-life of DB820 for all monkeys (Figure 6). DB844 was detected in CSF 1 h post dose, with a geometric mean (90% CI) concentration of 17 (7.3–39) nM, and was not detected thereafter. DB820 was not detected in the CSF, with only 2/7 monkeys (monkeys 571 and 624) showing sporadic low concentration (<4 nM) between 24 and 96 h post dose.

**Table 4:** Pharmacokinetics of DB844 and DB820 in vervet monkeys after the 14^th^ oral dose at 6 mg/kg (n = 7).

Outcome	Units	DB844		DB820	
AUC(0-∞)	nmol/L·day	70	(16, 290)	NC*	NC
AUC_last_	nmol/L·day	68	(16, 300)	1400	(760, 2500)
Metabolite: Parent AUC Ratio	NA	–	–	3	(1.1, 6.5)
Cl/F	L/day/kg	240	(56, 1000)	NC	NC
C_max_	nmol/L	430	(100, 1800)	190	(110, 320)
t_1/2_	day	0.24	(0.14, 0.40)	NC†	NC
T_max_	day	0.04	(0.04, 0.17)	0.17	(0.08, 0.17)

Key: Values are geometric mean (90% CI) except for metabolite∶parent AUC ratio and T_max_, which are reported as median (range). Metabolite∶parent AUC ratio was derived using the AUC from time zero to the last common time point for DB844 and DB820.;

*: % AUC extrapolated to infinite time >25%.

**†:** Duration of sample collection was insufficient to derive an accurate estimate of terminal half-life; AUC(0-∞), AUC from zero to infinite time; AUC_last_, AUC from time zero to the last measurable concentration; Cl/F, apparent (oral) clearance; C_max_, maximum concentration; t_1/2_, terminal half-life; T_max_, time to reach C_max_; NA, not applicable; NC, not calculable.

## Discussion

The current study has shown that the novel diamidine prodrug (DB844) was effectively metabolized by male vervet monkey liver microsomes to yield at least seven metabolites which were also detected when DB844 was incubated with human liver microsomes [13]. The order in which metabolites were generated in the monkey liver microsomal/drug mixtures and their relative concentrations, dominated by M1A (DB1284) and M1B (DB1058) within the first 20 minutes and by M3 (DB821) thereafter, were also similar to the pattern observed in human liver microsomes [13] suggesting that vervet monkeys would be a useful animal model for evaluation of a drug (DB844) that was in development as a potential therapeutic agent for a human disease (HAT). Our study did not investigate the enzymes responsible for the conversion of DB844 to DB820. It has however been previously shown that conversion of pafuramidine (DB289) to furamidine (DB75) was catalysed by cytochrome P450 enzymes and cytochrome b_5_/b_5_ reductase in the human liver [22], [29]. Liver microsomes derived from female vervet monkeys and Cynomolgus monkeys metabolized DB844 as efficiently as those derived from male vervets (data not shown), thus justifying evaluation of DB844 in monkeys.

The prodrug (DB844) was well tolerated when tested in uninfected vervet monkeys at the lowest dose (5 mg/kg×10 days) but was toxic to both monkeys when administered at the highest dose (20 mg/kg×8 days). The middle dose (10 mg/kg×10 days) was well tolerated by 1/2 monkeys, suggesting that this dose was slightly more than the maximum tolerated dose in uninfected monkeys. Clinical signs of overt toxicity were detected either after completion of dose regimen (10 mg/kg group) or late into the treatment regimen (20 mg/kg group), suggesting that overt toxicity was dependent on both the daily drug dose and duration of drug administration. These results were consistent with a previous report in which several pentamidine derivatives were associated with acute to chronic toxicity in rodents, which was cumulative in nature with respect to drug exposure [30]. The no observed adverse effects level (NOAEL) for oral DB844 in un-infected monkeys was therefore in the range 5–<10 mg/kg, and as a result, daily dose levels of 5 and 6 mg/kg were chosen for the subsequent efficacy study.

DB844/DB820 concentrations in plasma samples from un-infected monkeys (5 mg/kg group), though limited in scope, demonstrated that the prodrug was absorbed after oral administration and converted to the active metabolite (DB820). Similarly, when Cynomolgus monkeys were dosed orally with DB844 at 3 or 10 mg/kg, the drug was absorbed and the resulting plasma DB844/DB820 concentration-time profiles were comparable to those achieved in mice that were dosed at 25 and 100 mg/kg, respectively (Michael Z. Wang, personal communication). These findings were significant since DB844 at 25 or 100 mg/kg was subsequently determined to be curative for 1^st^ and 2^nd^ stage experimental murine HAT infections respectively [11], suggesting that comparatively low dose levels could be efficacious in primates. A period of dosing of 10–14 days was selected for the vervet DB844 efficacy study, partly informed by a report that during clinical trials of the related 1^st^ stage investigational HAT drug DB289, the duration of dosing had to be increased from 5 to 10 days in order to increase efficacy (Sonja Bernhard, Personal communication). In addition, the fact that in humans 2^nd^ stage HAT is treated for 10 days with melarsoprol or NECT) and 14 days with intravenous eflornithine [4] was considered since these dosing periods are partly influenced by the tissue invasive nature of the human infective parasites.

Oral DB844 achieved up to 43% cure rate in the vervet monkey model of 2^nd^ stage HAT. Prior to initiation of treatment at 28 DPI, all 16 monkeys in this study were confirmed to have trypanosomes and pathological white cell numbers in their CSF, confirming that the model fulfilled the criteria for classification of CNS (late, 2^nd^) stage disease [4]. Pathophysiology studies have shown that trypanosome entry into the CSF initiates meningitis and leads to elevated CSF nitric oxide and IgM concentrations, all further indicating CNS disease [31], [32]. Although occurrence of histologically demonstrable meningoencephalitis may not be guaranteed in the course of primary *T. b. rhodesiense* infections [31], [33], previous studies have shown that when treatment was initiated at 28 DPI in this monkey model, pafuramidine (DB289) and pentamidine did not cure any monkey ([12]; unpublished TRC-KARI data). The data reported in this study, therefore, indicates that DB844 had an improved activity in the CNS stage monkey model compared to the other diamidines.

Biological activity of orally administered chemotherapeutic agents against tissue invasive parasites such as *T. b. rhodesiense* is dependent upon absorption in the gut and attainment of effective exposure levels of the prodrug and/or active metabolites in body fluids and tissues. Pharmacokinetic evaluation of efficacy study (6 mg/kg group) monkeys confirmed that marked DB844 concentration was attained in plasma within 1 hr of the last dose. Peak plasma levels of DB820 were attained within 4 h post last dosing showing that metabolic conversion of DB844 to DB820 was comparable to previous observations in rats and cynomolgus monkeys involving the related prodrug/active metabolite pair, DB289/DB75 [34]. The peak plasma concentrations of the active drug (DB820) were 37 times (190/5.2) higher than the IC_50_ for *T. b. rhodesiense* STIB900; the DB820 concentrations remained at least 19 times (100/5.2) higher than the IC_50_ for *T. b. rhodesiense* STIB900 for at least 48 h post dosing, suggesting that alternative dose regimens in which the drug was dosed once every two days may be sufficient for bloodstream trypanosomes. The plasma C_max_ achieved by DB844 in our study was significantly higher than the 30–35 nM C_max_ values obtained when DB289 was dosed orally to cynomolgus monkey at 5 mg/kg [34]), consistent with similar observations when both DB844 and DB289 were dosed to mice [35].

Detection of DB844 in the CSF indicated BBB penetration and was likely responsible for observed reduction in trypanosome densities in CSF of all monkeys to below the limit of detection for varying periods and eventual cure of 3/7 (43%) group II monkeys. The active metabolite (DB820) was, however, detected only sporadically in CSF of two of the group II monkeys, one of which was eventually cured and one of which was not. The discordance between the CSF PK data and observed activity may result from three possibilities: i) No CSF samples were collected between 1–24 h post dosing. The reduced sampling time points, though justified due to ethical considerations, may have resulted in underestimation of DB820/DB844 in CSF; ii) Some of the intermediate metabolites of DB844, including the monoamidines DB1212, DB1285, M4A and M4B have been shown to have anti-trypanosmal activity in vitro [11]. Unfortunately, these were not quantified in the vervet CSF in our study; iii) trypanosomes were shown to be capable of accumulating DB820 and DB75 up to 15,000 times above mouse plasma concentrations [36], suggesting that trypanocidal drug concentrations could accumulate and reduce the density of trypanosomes in CSF despite low concentrations of the active metabolite/s.

Trypanosome recrudescence in the CSF preceded that in the blood (133 vs. 261 days), indicating that CSF/CNS remained a major sanctuary/source of relapse trypanosomes despite the improved efficacy of DB844 compared with other diamidines. This was consistent with the generally low CSF: plasma ratio of 1∶27 (3.7%) for DB844 at 1 h post last dosing. In addition, and in spite of the limitations highlighted above, that DB820 was hardly quantified in CSF while it was detected in plasma in relatively high concentrations, accounts for the observation that trypanosomes were eliminated more rapidly from the blood than from the CSF (data not shown). An alternative dosing regimen in which higher daily DB844 doses were administered once every two days as discussed above could possibly result into more DB844/DB820 crossing the BBB and improve CNS activity as observed in the GVR 35 mouse model [11]. The rationale for higher daily drug doses would be based on the fact that drug transport across BBB is influenced by concentration dependent passive diffusion, presence of efflux aiding P-glycoprotein transporters or multidrug resistance-associated protein transporter [37], [38]. Importantly, however, structure activity relationship studies need to be continued to identify molecules with superior activity against second stage HAT.

No DB844-related haematological aberrations were observed in the infected monkey model, indicating that low doses (5 and 6 mg/kg) were safe. Erythrocyte and platelet associated parameters recovered rapidly indicating that erythropoesis and thrombocytogenesis remained robust. White blood cells were similarly not adversely affected, suggesting that DB844 was not myelotoxic. However, trypanosome-induced anaemia, thrombocytopenia and leucopoenia were observed. These are common features of experimental *T.b. rhodesiense* infections in monkeys [12], [21], [39] and natural HAT infections in humans [40]–[42] whose severity is determined by parasite virulence, time lag from infection to therapeutic intervention and individual host differences. The haematology changes before treatment were comparable to those reported in previous infections with the KETRI2537 stabilate, indicating good reproducibility of the monkey model. Furthermore, resolution of haematology aberrations such as haemoglobin concentration and RDW was clearly related to treatment showing that these were additional indicators of therapeutic efficacy. In un-infected monkeys, the low dose regimen (5 mg/kg×10 days) similarly did not manifest haematologic toxicity. At high doses (>10 mg/kg), however, bleeding and haemosiderosis were observed in multiple organs possibly due to damage of endothelial membranes, increased sequestration of damaged erythrocytes and their subsequent destruction by tissue macrophages [43], [44].

Clinical chemistry results (group II, efficacy study) showed modest (2–3 fold) increases in plasma transaminases (AST and ALT) but not alkaline phosphatase, consistent with hepatocellular pathology. Importantly, these elevations in transaminases were reversible within 4–7 days after the last drug dose, indicating that they were likely caused by transient changes in the permeability of hepatocyte cell membranes (rather than necrosis). In contrast high doses (>10 mg/kg) caused significant hepatotoxicity that was mainly characterized by fatty degeneration (steatosis), focal necrosis, mononuclear infiltration and haemorrhages. Steatosis is a common toxicity of many other drugs, including tetracyclines, corticosteroids, non-steroidal anti-inflammatory drugs and diamidines [45]–[48]. Steatosis in the un-infected monkeys was likely caused by impairment of mitochondrial fatty acid ß-oxidation causing microvesicular steatosis and resulting in accumulation of lipid vesicles in the cytoplasm of hepatocytes as previously reported for other drugs [45]–[48].

Analysis of clinical chemistry indicators of renal pathology in efficacy group II (6 mg/kg) monkeys revealed that blood urea nitrogen (BUN) was minimally elevated while plasma creatinine was not, indicating that kidney function was only transiently affected. In contrast, histopathology results from the un-infected monkeys revealed evidence of renal tubular degeneration in the 10 and 20 mg/kg, showing that a more significant renal pathology resulted from the higher drug doses. Similarly, no lesions were seen at low doses in the GIT while moderate to severe gastroenteritis were observed at high doses. Overall therefore, toxicity was dose-dependent and, taken together with results of the efficacy study, indicated that there was a lack of therapeutic window for DB844. As a result of toxicity, alternative dose regimens to improve efficacy were not attempted.

In summary, this study showed that the prodrug DB844 achieved a moderate cure rate in the vervet monkey model of 2^nd^ stage HAT, which was in contrast with studies in mice in which much higher DB844 doses (ie 100 mg/kg×5 days) were tolerated and cured all 5/5 mice [11]. This is perhaps the main reason why new chemical entities (NCEs) that are targeted against HAT are preferably evaluated in both rodent and monkey models to obtain a more comprehensive understanding of safety, efficacy and pharmacokinetics before being forwarded for clinical trials in man. Although further development of DB844 against 2^nd^ stage HAT was discontinued due to the lack of a therapeutic window, DB844 has demonstrated that structural modifications of amidines could eventually result in molecules with promising CNS activity at tolerated dose levels. Indeed, novel amidine analogues with better efficacy and safety profiles have been identified and are currently being evaluated in this vervet monkey model of HAT, which will be reported in due course.

## Supporting Information

Figure S1 **Individual monkey activity/concentration-time profiles of aspartate amino transferase and blood urea nitrogen in plasma.** The monkeys were treated with DB844 at 6 mg/kg×14 days, from 28–41 days post infection with *T.b. rhodesiense* KETRI2537.
(TIF)

Figure S2 **Individual monkey concentration-time profiles of DB844 and DB820 in plasma.** The monkeys were treated orally with DB844 at 6 mg/kg×14 days, from 28–41 days post infection with *T.b. rhodesiense* KETRI2537. The insert graph shows the extended profiles up to 28 days post the last daily dose of DB844.
(TIF)
