# Supplementary figures and images for: Pharmacology of DB844, an Orally Active aza Analogue of Pafuramidine, in a Monkey Model of Second Stage Human African Trypanosomiasis
Source: PLoS Negl Trop Dis. 2012 Jul 24;6(7):e1734. doi: 10.1371/journal.pntd.0001734 (PMC3404106; doi:10.1371/journal.pntd.0001734)

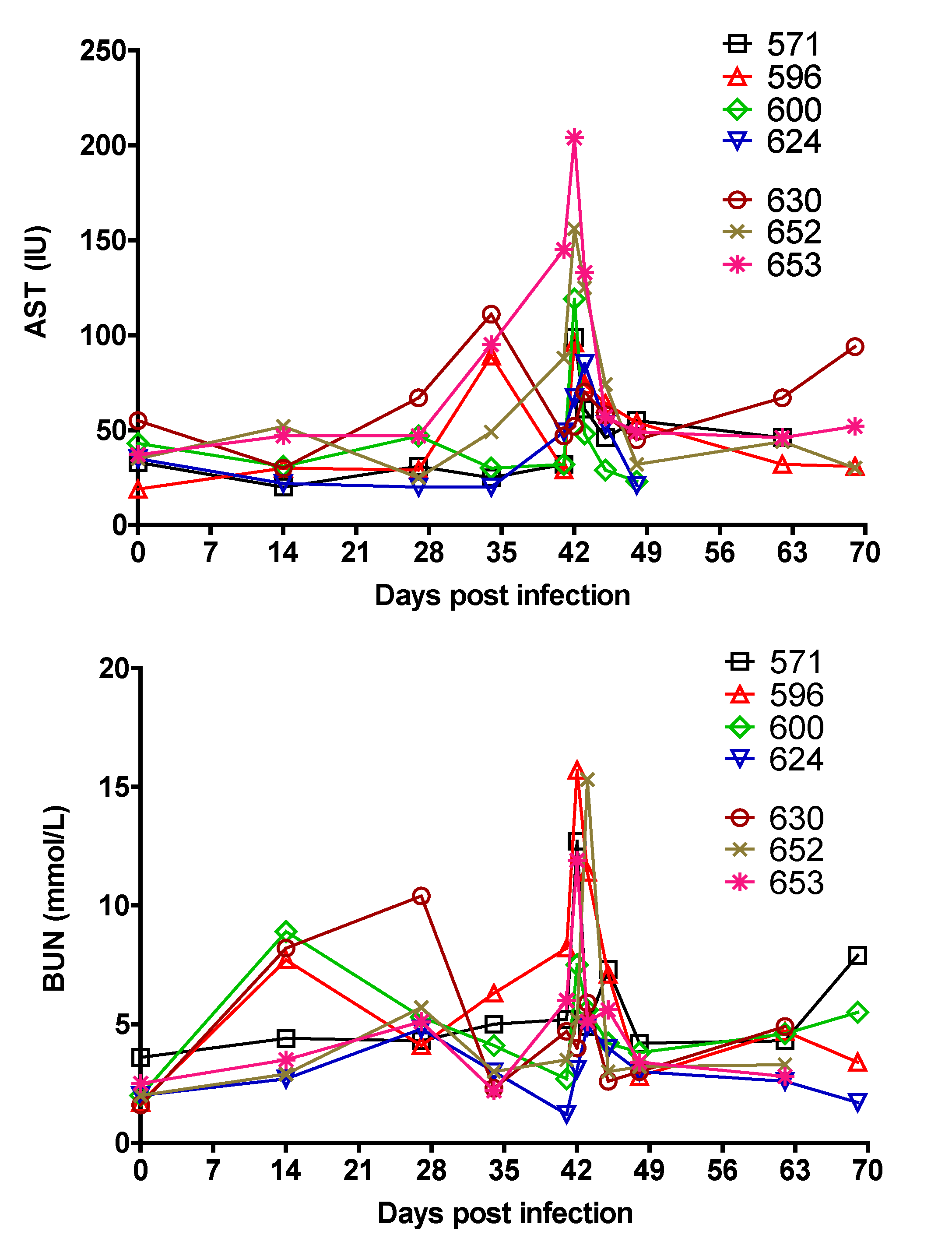

Supplement: Figure S1 — Individual monkey activity/concentration-time profiles of aspartate amino transferase and blood urea nitrogen in plasma. The monkeys were treated with DB844 at 6 mg/kg×14 days, from 28–41 days post infection with T.b. rhodesiense KETRI2537. (TIF) [file pntd.0001734.s001.tif]

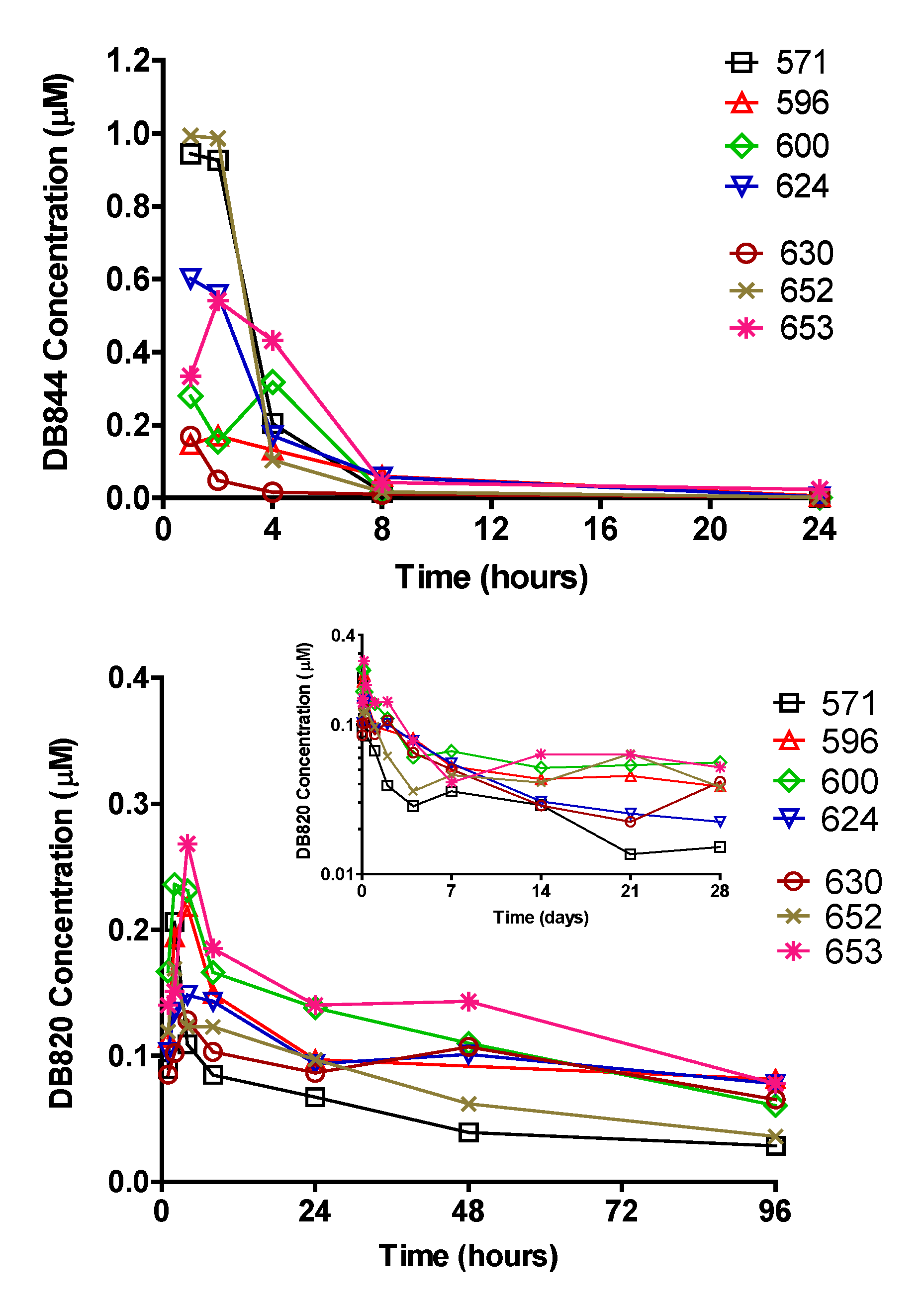

Supplement: Figure S2 — Individual monkey concentration-time profiles of DB844 and DB820 in plasma. The monkeys were treated orally with DB844 at 6 mg/kg×14 days, from 28–41 days post infection with T.b. rhodesiense KETRI2537. The insert graph shows the extended profiles up to 28 days post the last daily dose of DB844. (TIF) [file pntd.0001734.s002.tif]
